# Variation of Anxiety and Depression During a 3-Year Period as Well as Their Risk Factors and Prognostic Value in Postoperative Bladder Cancer Patients

**DOI:** 10.3389/fsurg.2022.893249

**Published:** 2022-07-19

**Authors:** Meiling Guo, Yanjie Li, Wentao Wang, Xu Kang, Guiyun Chen

**Affiliations:** ^1^ Department of Urology Surgery, Harbin Medical University Cancer Hospital, Harbin, China; ^2^ Heilongjiang Province Public Security Department Ankang Hospital, Addiction Treatment Centre, Harbin, China; ^3^ Medical Department, Harbin Medical University Cancer Hospital, Harbin, China; ^4^ Nursing Department, Harbin Medical University Cancer Hospital, Harbin, China

**Keywords:** anxiety and depression, bladder cancer, longitudinal change, risk factors, survival

## Abstract

**Background:**

Anxiety and depression are commonly recognized and prognostically relevant in cancer patients. The aim of this study was to explore the 3-year longitudinal changes in anxiety and depression, their risk factors, and prognostic value in patients with bladder cancer.

**Methods:**

Hospital Anxiety and Depression Scale for anxiety (HADS-A) and depression (HADS-D) scores of 120 postoperative bladder cancer patients and 100 healthy controls (HCs) were assessed. Additionally, the HADS-A and HADS-D scores of bladder cancer patients were determined at 1 year, 2 years, and 3 years post surgery.

**Results:**

HADS-A score (7.7 ± 3.0 vs. 4.8 ± 2.6), anxiety rate (38.3% vs. 9.0%), HADS-D score (7.7 ± 3.3 vs. 4.3 ± 2.6), depression rate (40.0% vs. 11.0%), as well as anxiety degree and depression degree, were all increased in bladder cancer patients compared with HCs (all *P *< 0.001). Besides, the HADS-A score gradually increased from baseline to 3 years (*P *= 0.004), while the anxiety rate, HADS-D score, and depression rate did not change significantly (all *P *> 0.050). Gender, tumor size, marriage status, hypertension, diversity, and lymph node (LN) metastasis were associated with anxiety or depression in patients with bladder cancer (all *P *< 0.050). Anxiety was associated with shortened overall survival (OS) (*P *= 0.024) but did not link with disease-free survival (DFS) (*P *= 0.201); depression was not correlated with either DFS or OS (both *P *> 0.050).

**Conclusion:**

The prevalence and severity of anxiety and depression are high in patients with bladder cancer, which are influenced by gender, tumor features, marriage status, and hypertension; in addition, their correlation with survival is relatively weak.

## Introduction

Bladder cancer is the 10th most common cancer worldwide, with 570,000 new cases and 210,000 deaths in 2020 (1–3). Transurethral resection of bladder tumor (TURBT) is the gold standard for definitive diagnosis and the standard treatment for non-muscle-invasive bladder cancer (NMIBC), while radical cystectomy with lymph node dissection is the primary surgical regimen for patients with muscle-invasive or advanced bladder cancer (4–6). Although the 5-year survival for patients with resectable bladder cancer is estimated to be between 35% and 69%, many patients will encounter postoperative complications (such as urinary tract infection, frequent urination, cachexia, etc.) after surgeries; meanwhile, they will also suffer from mental disorders (including anxiety, depression, suicidality, etc.), which will ultimately impair their quality of life (7–10).

Anxiety and depression are the most common psychological problems observed in patients with postoperative bladder cancer and have been frequently reported in previous studies (11–14). For instance, a systematic review exhibited that the prevalence of anxiety (ranges from 12.5% to 71.3%) and depression (ranges from 4.7% to 78%) in bladder cancer patients varies in different regions and countries (11). Another study disclosed that more than half of bladder cancer patients were recognized with anxiety and depression after treatment; meanwhile, mental and psychological disorders led to poor clinical outcomes (12). However, most relevant studies were cross-sectional or had a short follow-up duration (less than 1 year), let alone the long-term follow-up.

Hence, this study detected the anxiety and depression status during a 3-year follow-up period with the aim of exploring their longitudinal changes, risk factors, and predictive value for survival in patients with bladder cancer.

## Methods

### Subjects

This prospective study serially included bladder cancer patients who underwent tumor resection by TURBT or radical cystectomy (by means of open surgery, laparoscope, or robot-assisted laparoscope) from January 2016 to March 2018; the surgical regimens depended on patient's pathological type (NMIBC or MIBC). The inclusion criteria were as follows: (1) pathologically diagnosed with bladder cancer; (2) more than 18 years old; (3) received TURBT or radical cystectomy; (4) had no difficulty in completing the evaluation of anxiety and depression using the Hospital Anxiety and Depression Scale (HADS); (5) willing to comply with the follow-up schedule. The patients who met the following conditions were excluded: (1) having complications with a severe mental disorder, such as bipolar disorder and schizophrenia; (2) having severe cognitive impairment; (3) who were concomitant with other malignancies; (4) who were pregnant or lactating women. Besides, during the same period, healthy subjects who came to the hospital for physical examinations were enrolled in the study as health controls (HCs). The inclusion criteria for HCs were: (1) having no abnormities in physical examinations; (2) having matched age and gender to bladder cancer patients; (3) having no severe mental disorders or severe cognitive impairments; (4) those without a prior history of cancers or other malignant diseases; (5) those who were non-pregnant and non-lactating. The exclusion criteria for bladder cancer patients were also suitable for HCs. The study was permitted by the Ethics Committee. All patients in this study signed the informed consent.

### Assessment of Anxiety and Depression

The HADS for anxiety (HADS-A) score and the HADS for depression (HADS-D) score were used to access the anxiety status and depression status of all subjects, respectively. For patients with bladder cancer, the assessments were carried out on the day of discharge from the hospital (baseline), at 1 year (±1 month) after surgery, 2 years (±1 month) after surgery, and 3 years (±1 month) after surgery. For HCs, the assessments were performed after enrollment. The maximum score of HADS-A or HADS-D was 21, with a higher score indicating a severer anxiety or depression status. Specifically, the HADS-A or HADS-D was divided into four grades: 0–7, no anxiety or no depression; 8–10, mild status; 11–14, moderate status; 15–21, severe status (15).

### Evaluation of Survival Data

All patients with bladder cancer were followed up through outpatient visits for at least 3 years until March 2021. During the follow-up period, the disease status of patients was recorded. Totally, 23 (19.2%) patients were lost to follow-up, and 14 (11.2%) patients died. Based on the follow-up data, disease-free survival (DFS), overall survival (OS), and cancer-specific survival (CSS) were imputed.

### Statistical Analysis

Statistical analysis and figure plotting were performed using SPSS V.22.0 (IBM Corp., USA) and GraphPad Prism V.6.0 (GraphPad Software Inc., USA), respectively. Differences in anxiety and depression status between the two groups were analyzed using the Student's *t*-test, Mann–Whitney *U* test, or Chi-square test. Changes in anxiety and depression status over time were determined using the repeated ANOVA test or Cochran's *Q* test. Correlations of clinical features with anxiety and depression status were assessed using the Chi-square test or Fisher's exact test. Independence risk factors of anxiety status and depression status were determined using forward-stepwise multivariate logistic regression analysis with all potential parameters included. Correlation of DFS, OS, and CSS with anxiety and depression status was presented using the Kaplan–Meier method and evaluated using the log-rank test. A score of *P *< 0.05 was considered significant.

## Results

### Clinical Features of Bladder Cancer Patients

In this study, a total of 120 bladder cancer patients and 100 HCs were recruited. The mean age of the 120 patients with bladder cancer was 61.6 ± 10.4 years, of whom 27 (22.5%) were females, and 93 (77.5%) were males (Table 1). Moreover, the median tumor size was 2.5 (interquartile range (IQR): 2.0–3.0) cm; furthermore, 84 (70.0%) patients were diagnosed with a single tumor, whereas the remaining 36 (30.0%) patients were diagnosed with multiple tumors. In terms of the tumor stage, 82 (68.3%) patients were defined as Ta–T1 stage, and 38 (31.7%) patients were assessed as T2–T4 stage. Furthermore, 11 (9.2%) patients were found to have lymph node (LN) metastasis. In terms of the pathological grade, 75 (62.5%) patients were classified as low grade; meanwhile, 45 (37.5%) patients were classified as high grade. With regard to the surgery type, 78 (65.0%), 36 (30.0%), and 6 (5.0%) patients received TURBT, radical cystectomy by laparoscopic surgery, and radical cystectomy by open surgery, accordingly. Additionally, the detailed clinical features of the patients with bladder cancer are listed in Table 1.

**Table 1:** Clinical features of bladder cancer patients.

Items	Bladder cancer patients (*N* = 120)
Age (years), mean ± SD	61.6 ± 10.4
Gender, No. (%)	
Female	27 (22.5)
Male	93 (77.5)
Smoker, No. (%)	51 (42.5)
Drinker, No. (%)	37 (30.8)
Marriage status, No. (%)	
Married	92 (76.7)
Single/divorced/widowed	28 (23.3)
Employment status before surgery, No. (%)	
Employed	43 (35.8)
Unemployed	77 (64.2)
Level of education, No. (%)	
Primary school or less	10 (8.3)
High school	52 (43.3)
Undergraduate	40 (33.3)
Graduate or above	18 (15.0)
Location, No. (%)	
Urban	104 (86.7)
Rural	16 (13.3)
Hypertension, No. (%)	46 (38.3)
Hyperlipidemia, No. (%)	30 (25.0)
Diabetes, No. (%)	15 (12.5)
Tumor size (cm), median (IQR)	2.5 (2.0–3.0)
Multiplicity, No. (%)	
Single	84 (70.0)
Multiple	36 (30.0)
Tumor stage, No. (%)	
Ta–T1	82 (68.3)
T2–T4	38 (31.7)
LN metastasis, No. (%)	11 (9.2)
Pathological grade, No. (%)	
Low grade	75 (62.5)
High grade	45 (37.5)
Surgery type, No. (%)	
TURBT	78 (65.0)
Radical cystectomy by laparoscopic surgery	36 (30.0)
Radical cystectomy by open surgery	6 (5.0)

*SD, standard deviation; IQR, interquartile range; LN, lymph node; TURBT, transurethral resection of bladder tumor*.

### Comparison of Anxiety and Depression Status Between Bladder Cancer Patients and HCs

Patients with bladder cancer exhibited aggravated anxiety and depression compared with HCs (Table 2). More specifically, both the mean HADS-A scores (7.7 ± 3.0 vs. 4.8 ± 2.6) and the anxiety rates (38.3% vs. 9.0%) were elevated in bladder cancer patients compared with HCs (both *P *< 0.001). Besides, anxiety was more severe in patients with bladder cancer than in HCs (*P *< 0.001). Similarly, the mean HADS-D scores (7.7 ± 3.3 vs. 4.3 ± 2.6) and depression rates (40.0% vs. 11.0%) were increased in patients with bladder cancer than in HCs (both *P *< 0.001). Also, depression was more severe in patients with bladder cancer than in HCs (*P *< 0.001).

**Table 2:** Comparison of anxiety and depression status between bladder cancer patients and HCs.

Items	Bladder cancer patients (*N* = 120)	HCs (*N* = 100)	*P* value
HADS-A score, mean ± SD	7.7 ± 3.0	4.8 ± 2.6	**<0**.**001**
Anxiety rate, No. (%)	46 (38.3)	9 (9.0)	**<0**.**001**
Anxiety degree, No. (%)			**<0**.**001**
No anxiety	74 (61.7)	91 (91.0)	** **
Mild anxiety	23 (19.2)	7 (7.0)	** **
Moderate anxiety	19 (15.8)	2 (2.0)	** **
Severe anxiety	4 (3.3)	0 (0.0)	** **
HADS-D score, mean ± SD	7.7 ± 3.3	4.3 ± 2.6	**<0**.**001**
Depression rate, No. (%)	48 (40.0)	11 (11.0)	**<0**.**001**
Depression degree, No. (%)			**<0**.**001**
No depression	72 (60.0)	89 (89.0)	** **
Mild depression	25 (20.8)	9 (9.0)	** **
Moderate depression	17 (14.2)	2 (2.0)	** **
Severe depression	6 (5.0)	0 (0.0)	** **

*HCs, health controls; HADS-A, Hospital Anxiety and Depression Scale-Anxiety; SD, standard deviation; HADS-D, Hospital Anxiety and Depression Scale-Depression.*

### Longitudinal Changes of Anxiety and Depression in Bladder Cancer Patients

**Figure 1:**
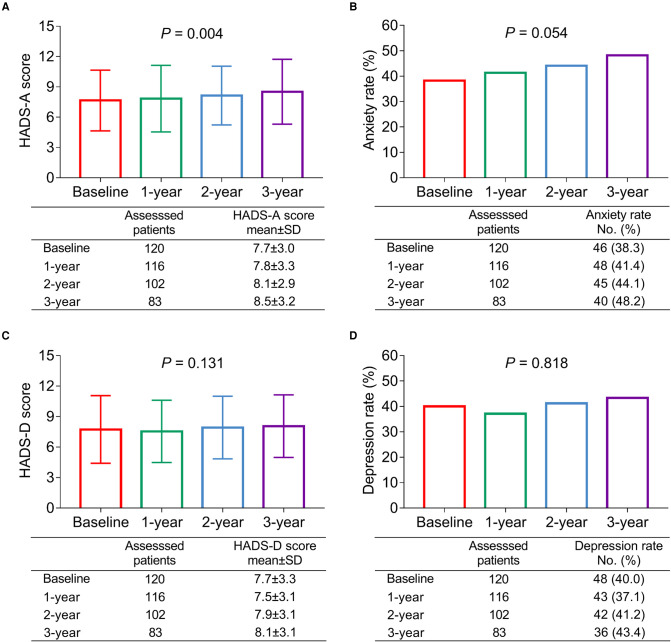
Anxiety gradually aggravated, while depression status was not changed from baseline to 3 years in patients with bladder cancer. The longitudinal changes of the HADS-A score (**A**); anxiety rate (**B**); HADS-D score (**C**); and depression rate (**D**) during 3-year follow-up duration in patients with bladder cancer.

The HADS-A scores of patients with bladder cancer gradually increased from baseline to 3 years (*P *= 0.004, Figure 1A); meanwhile, the anxiety rate also disclosed an increasing trend from baseline to 3 years (but lacked statistical significance) (*P *= 0.054, Figure 1B). In detail, the HADS-A scores at baseline, 1, 2, and 3 years were 7.7 ± 3.0, 7.8 ± 3.3, 8.1 ± 2.9, and 8.5 ± 3.2, respectively; meanwhile, the anxiety rates at baseline, 1, 2, and 3 years were 38.3%, 41.4%, 44.1%, and 48.2%, correspondingly. There was no difference in HADS-D scores (*P *= 0.131, Figure 1C) at baseline, 1, 2, and 3 years in patients with bladder cancer, as well as in the depression rate (*P *= 0.818, Figure 1D). In detail, the HADS-D scores at baseline, 1, 2, and 3 years were 7.7 ± 3.3, 7.5 ± 3.1, 7.9 ± 3.1, and 8.1 ± 3.1, respectively; meanwhile, the depression rates at baseline, 1, 2, and 3 years were 40.0%, 37.1%, 41.2%, and 43.4%, correspondingly.

### Correlation of Clinical Features with Anxiety and Depression in Bladder Cancer Patients

Female (vs. male) (*P *= 0.037), tumor size ≥3 cm (vs. <3 cm) (*P *= 0.044), multiple tumors (vs. single) (*P *= 0.033), and LN metastasis (vs. absent) (*P *= 0.003) were associated with elevated anxiety rate in patients with bladder cancer (Table 3). However, age, smoking, drinking, marriage status, employment status before surgery, level of education, location, hypertension, hyperlipidemia, diabetes, tumor stage, and pathological grade were not linked with anxiety (all *P *> 0.050).

**Table 3:** Correlation of clinical features with anxiety status among bladder cancer patients.

Items	Anxiety	No anxiety	*P* value
Age, No. (%)			0.161
<60 years	14 (30.4)	32 (69.6)	
≥60 years	32 (43.2)	42 (56.8)	
Gender, No. (%)			**0**.**037**
Female	15 (55.6)	12 (44.4)	
Male	31 (33.3)	62 (66.7)	
Smoker, No. (%)			0.864
No	26 (37.7)	43 (62.3)	
Yes	20 (39.2)	31 (60.8)	
Drinker, No. (%)			0.252
No	29 (34.9)	54 (65.1)	
Yes	17 (45.9)	20 (54.1)	
Marriage status, No. (%)			0.147
Married	32 (34.8)	60 (65.2)	
Single/divorced/widowed	14 (50.0)	14 (50.0)	
Employment status before surgery, No. (%)			0.173
Employed	13 (30.2)	30 (69.8)	
Unemployed	33 (42.9)	44 (57.1)	
Level of education, No. (%)			0.282
Primary school or less	3 (30.0)	7 (70.0)	
High school	21 (40.4)	31 (59.6)	
Undergraduate	12 (30.0)	28 (70.0)	
Graduate or above	10 (55.6)	8 (44.4)	
Location, No. (%)			0.632
Urban	39 (37.5)	65 (62.5)	
Rural	7 (43.8)	9 (56.3)	
Hypertension, No. (%)			0.194
No	25 (33.8)	49 (66.2)	
Yes	21 (45.7)	25 (54.3)	
Hyperlipidemia, No. (%)			0.515
No	33 (36.7)	57 (63.3)	
Yes	13 (43.3)	17 (56.7)	
Diabetes, No. (%)			0.065
No	37 (35.2)	68 (64.8)	
Yes	9 (60.0)	6 (40.0)	
Tumor size, No. (%)			**0**.**044**
<3 cm	29 (33.0)	59 (67.0)	** **
≥3 cm	17 (53.1)	15 (46.9)	** **
Multiplicity, No. (%)			**0**.**033**
Single	27 (32.1)	57 (67.9)	
Multiple	19 (52.8)	17 (47.2)	
Tumor stage, No. (%)			0.074
Ta–T1	27 (32.9)	55 (67.1)	
T2–T4	19 (50.0)	19 (50.0)	
LN metastasis, No. (%)			**0**.**003**
Absent	37 (33.9)	72 (66.1)	
Present	9 (81.8)	2 (18.2)	
Pathological grade, No. (%)			0.065
Low grade	24 (32.0)	51 (68.0)	
High grade	22 (48.9)	23 (51.1)	

*LN, lymph node*.

Besides, single/divorced/widowed (vs. married) (*P *= 0.034), hypertension (vs. no) (*P *= 0.032), multiple tumors (vs. single) (*P *= 0.023), and LN metastasis (vs. absent) (*P *= 0.026) were correlated with an increased depression rate in patients with bladder cancer (Table 4). Nevertheless, age, gender, smoking, drinking, employment status before surgery, level of education, location, hyperlipidemia, diabetes, tumor size, tumor stage, and pathological grade were not related to depression (all *P *> 0.050).

**Table 4:** Correlation of clinical features with depression status among bladder cancer patients.

Items	Depression	No depression	*P* value
Age, No. (%)			0.592
<60 years	17 (37.0)	29 (63.0)	
≥60 years	31 (41.9)	43 (58.1)	
Gender, No. (%)			0.153
Female	14 (51.9)	13 (48.1)	
Male	34 (36.6)	59 (63.4)	
Smoker, No. (%)			0.327
No	25 (36.2)	44 (63.8)	
Yes	23 (45.1)	28 (54.9)	
Drinker, No. (%)			0.628
No	32 (38.6)	51 (61.4)	
Yes	16 (43.2)	21 (56.8)	
Marriage status, No. (%)			**0**.**034**
Married	32 (34.8)	60 (65.2)	
Single/divorced/widowed	16 (57.1)	12 (42.9)	
Employment status before surgery, No. (%)			0.214
Employed	14 (32.6)	29 (67.4)	
Unemployed	34 (44.2)	43 (55.8)	
Level of education, No. (%)			0.626
Primary school or less	5 (50.0)	5 (50.0)	
High school	23 (44.2)	29 (55.8)	
Undergraduate	13 (32.5)	27 (67.5)	
Graduate or above	7 (38.9)	11 (61.1)	
Location, No. (%)			0.742
Urban	41 (39.4)	63 (60.6)	
Rural	7 (43.8)	9 (56.3)	
Hypertension, No. (%)			**0**.**032**
No	24 (32.4)	50 (67.6)	
Yes	24 (52.2)	22 (47.8)	
Hyperlipidemia, No. (%)			0.667
No	35 (38.9)	55 (61.1)	
Yes	13 (43.3)	17 (56.7)	
Diabetes, No. (%)			0.091
No	39 (37.1)	66 (62.9)	
Yes	9 (60.0)	6 (40.0)	
Tumor size, No. (%)			0.354
<3 cm	33 (37.5)	55 (62.5)	
≥3 cm	15 (46.9)	17 (53.1)	
Multiplicity, No. (%)			**0**.**023**
Single	28 (33.3)	56 (66.7)	
Multiple	20 (55.6)	16 (44.4)	
Tumor stage, No. (%)			0.128
Ta–T1	29 (35.4)	53 (64.6)	
T2–T4	19 (50.0)	19 (50.0)	
LN metastasis, No. (%)			**0**.**026**
Absent	40 (36.7)	69 (63.3)	
Present	8 (72.7)	3 (27.3)	
Pathological grade, No. (%)			0.248
Low grade	27 (36.0)	48 (64.0)	
High grade	21 (46.7)	24 (53.3)	

*LN, lymph node*.

Further, forward-stepwise multivariate logistic regression analyses were conducted to investigate the independent risk factors of anxiety and depression in bladder cancer patients. Diabetes (vs. no) (odds ratio (OR): 3.528, *P *= 0.035), multiple tumors (vs. single) (OR: 2.612, *P *= 0.028), and LN metastasis (vs. absent) (OR: 10.252, *P *= 0.005) were independently linked with the occurrence of anxiety; meanwhile, multiple tumors (vs. single) (OR: 2.500, *P *= 0.028) and LN metastasis (vs. absent) (OR: 4.600, *P *= 0.034) were also independently associated with the occurrence of depression in bladder cancer patients (Supplementary Table 1).

With regard to the correlation between surgery type and HADS score among bladder cancer patients, it was observed that the HADS-A score at baseline was varied among patients who received TURBT, radical cystectomy by laparoscopic surgery, and radical cystectomy by open surgery (*P *= 0.029); in detail, the respective HADS-A score at baseline was highest in patients treated with radical cystectomy by open surgery (10.5 ± 1.5), followed by patients who received radical cystectomy by laparoscopic surgery (8.0 ± 3.2), and lowest in patients treated with TURBT (7.3 ± 2.9) (Supplementary Table 2). While the HADS-A score at 1, 2, and 3 years and the HADS-D score at baseline, 1, 2, and 3 years showed no difference among patients with different surgery types (all *P *< 0.050), they only disclosed a trend that they were highest in patients with radical cystectomy by open surgery, followed by patients with radical cystectomy by laparoscopic surgery, and lowest in patients with TURBT.

### Correlation of Anxiety and Depression at Baseline with Survival in Bladder Cancer Patients

**Figure 2:**
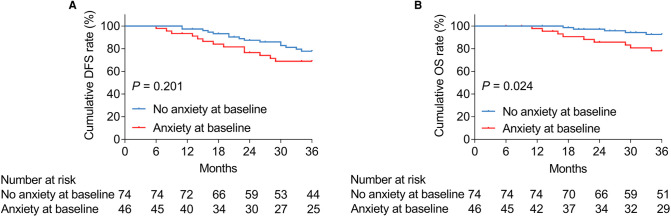
Anxiety at baseline was related to shortened OS in patients with bladder cancer. Correlation of anxiety at baseline with cumulative DFS (**A**) and OS (**B**) in patients with bladder cancer.

**Figure 3:**
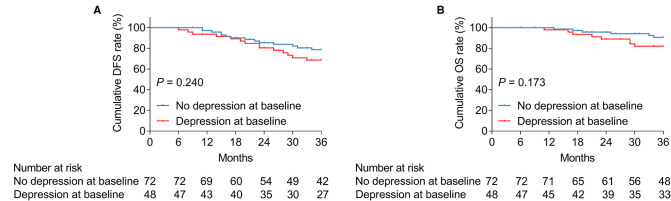
Depression at baseline was not linked with DFS or OS in patients with bladder cancer. Correlation of depression at baseline with cumulative DFS (**A**) and OS (**B**) in patients with bladder cancer.

Anxiety at baseline was not linked with cumulative DFS (*P *= 0.201, Figure 2A), whereas it was associated with shortened cumulative OS (*P *= 0.024, Figure 2B) in patients with bladder cancer. In terms of depression at baseline, it was correlated with neither cumulative DFS (*P *= 0.240, Figure 3A) nor OS (*P *= 0.173, Figure 3B) in bladder cancer patients.

Among 14 death cases, 12 cases were assessed as cancer-related deaths. Moreover, anxiety at baseline was related to reduced CSS in patients with bladder cancer (*P *= 0.025, Supplementary Figure 1A), whereas depression at baseline was not associated with CSS (*P *= 0.180, Supplementary Figure 1B).

## Discussion

There is a growing trend of bladder cancer patients to undergo psychological disorders (such as anxiety and depression) after surgical treatment, as has been demonstrated (13, 16–19). For instance, a previous study indicated that many bladder cancer patients who received radical cystectomy suffered from anxiety and depression during the perioperative period, with a prevalence rate of 34% (16). However, the previous studies were primarily cross-sectional, and only a few had a short-term follow-up period, let alone the long-term follow-up. The current study showed that their anxiety status, depression status, and incidence rates were all elevated in bladder cancer patients than HCs. Besides, there was an increasing trend of anxiety status from baseline to 3 years in bladder cancer patients, whereas there was no difference in depression. The possible reasons might be as follows: (1) Patients tend to panic about cancer and worry about their lives, and the negative emotions would cause anxiety and depression. (2) Patients with bladder cancer usually encounter postoperative complications (such as urinary tract infection, frequent urination, etc.) after surgical treatment, which would aggravate their anxiety and depression (19). Therefore, combining these two explanations, patients with bladder cancer have a higher prevalence and severity of anxiety and depression than HCs. (3) Those postoperative patients initially feel relieved after surgical treatment, while they would be worried about the recurrent over time, and then their anxiety would gradually increase during the 3-year follow-up period. Therefore, it is necessary to provide nursing, health education, and adaptability improvement for patients with recurrence to relieve them of anxiety and depression.

Apart from investigating the detailed anxiety and depression status of bladder cancer patients, this study found that the gender female (vs. male), tumor size ≥3 cm (vs. <3 cm), multiple tumors (vs. single), and LN metastasis (vs. absent) were associated with an elevated anxiety rate, whereas single/divorced/widowed (vs. married), hypertension (vs. no), multiple tumors (vs. single), and LN metastasis (vs. absent) were correlated with an increased depression rate. Probable explanations might be as follows: (1) It was observed that females were more likely to encounter anxiety during the hormonal flux period, whereas testosterone in males could be protective against anxiety (20, 21). Thus, females (vs. males) were correlated with an increased anxiety rate in patients with bladder cancer. (2) Single/divorced/widowed patients were more likely to feel lonely and unsupported, which would lead to depression (22, 23). Therefore, single/divorced/widowed was linked with depression in patients with bladder cancer. (3) The previous study showed that antihypertensive medication regimens might cause depression (24). Hence, hypertension was associated with depression in patients with bladder cancer. (4) Patients with a large tumor size, multiple tumors, or LN metastasis often suffered from limited treatment efficacy and worse clinical outcomes, which made them more anxious and depressed (25). Thus, tumor size ≥3 cm (vs. <3 cm) was related to elevated anxiety; meanwhile, multiple tumors (vs. single) and LN metastasis (vs. absent) were correlated with increased anxiety as well as depression in patients with bladder cancer. Additionally, the HADS-A score was highest in patients treated with radical cystectomy by open surgery, followed by patients who receive radical cystectomy by laparoscopic surgery, and lowest in patients treated with TURBT. While the HADS-A score at 1, 2, and 3 years, and the HADS-D score at baseline, 1, 2, and 3 years showed no difference among patients with different surgery types, they only disclosed a trend that they were highest in patients with radical cystectomy by open surgery, followed by patients with radical cystectomy by laparoscopic surgery, and lowest in patients with TURBT. The possible explanation for this might be as follows: Radical cystectomy by open surgery tended to cause more injuries than the other two surgery types, which would lead to more serious anxiety and depression, while its impairment was obvious only in a short term. Hence, the difference in long-term HADS-A and HADS-D scores among patients with different surgery types was not obvious.

In terms of the correlation of anxiety and depression with survival in bladder cancer patients, the previous studies exhibited that bladder cancer patients with post-treatment anxiety and depression tend to have worse OS and cancer-specific survival (9, 12). Unlike the present study, which found that anxiety at baseline was not associated with DFS but with shortened OS, depression at baseline was not correlated with either DFS or OS in patients with bladder cancer. The possible reason for this might be as follows: Anxiety and depression affected patients compliance with treatment to some extent, whereas the survival was more directly influenced by treatment efficacy (such as surgical treatment and pharmacotherapy, etc.), tumor stage, and other factors. Therefore, the correlation of anxiety and depression with survival in bladder cancer patients was relatively weak.

There were some limitations in the current study. First, the sample size was relatively small, which would result in weak statistical power. Second, the mental status of recurrent patients was usually worse than that of newly diagnosed patients, which deserved further study. Third, the anxiety and depression status of those patients prior to surgical treatment was undetected.

In conclusion, the prevalence and severity of anxiety and depression are high in patients with bladder cancer, which are influenced by gender, tumor features, marriage status, and hypertension; in addition, their correlation with survival is relatively weak.

## Data Availability

The original contributions presented in the study are included in the article/Supplementary Material, further inquiries can be directed to the corresponding author/s.
