# Supplementary figures and images for: Variation of Anxiety and Depression During a 3-Year Period as Well as Their Risk Factors and Prognostic Value in Postoperative Bladder Cancer Patients
Source: Front Surg. 2022 Jul 19;9:893249. doi: 10.3389/fsurg.2022.893249 (PMC9343671; doi:10.3389/fsurg.2022.893249)

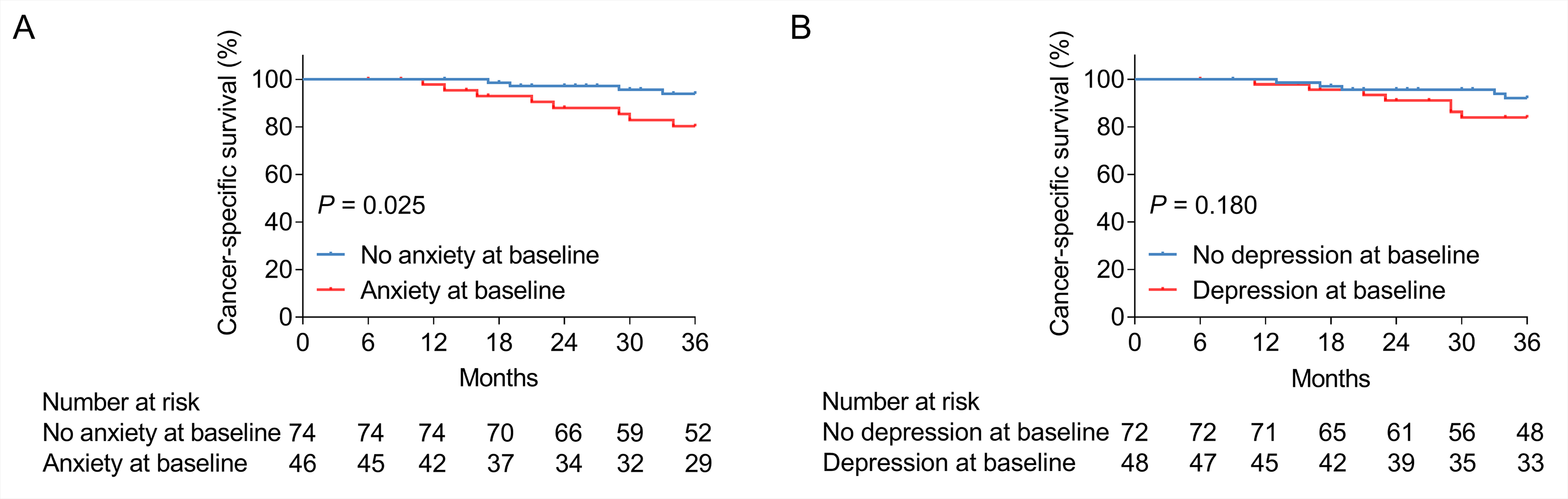

Supplement: Supplementary file 1 [file Image_1_v1.tif]
