# Supplementary material for: Variation of Anxiety and Depression During a 3-Year Period as Well as Their Risk Factors and Prognostic Value in Postoperative Bladder Cancer Patients
Source: Front Surg. 2022 Jul 19;9:893249. doi: 10.3389/fsurg.2022.893249 (PMC9343671; doi:10.3389/fsurg.2022.893249)
Supplement: Supplementary file 2 [file Table_5_v1.docx]

**Supplementary Table 1.** Multivariate logistic regression analysis for anxiety status and depression status.

| Items | *P* value | OR | 95% CI | |
| --- | --- | --- | --- | --- |
|  |  |  | Lower | Upper |
| **Forward-stepwise multivariate logistic regression analysis for anxiety status** | | | | |
| Diabetes (yes vs. no) | 0.035 | 3.528 | 1.094 | 11.378 |
| Multiplicity (multiple vs. single) | 0.028 | 2.612 | 1.109 | 6.152 |
| LN metastasis (present vs. absent) | 0.005 | 10.252 | 2.013 | 52.218 |
| **Forward-stepwise multivariate logistic regression analysis for depression status** | | | | |
| Multiplicity (multiple vs. single) | 0.028 | 2.500 | 1.105 | 5.655 |
| LN metastasis (present vs. absent) | 0.034 | 4.600 | 1.126 | 18.794 |

OR, odds ratio; CI, confidence interval; LN, lymph node.
