# Supplementary material for: Variation of Anxiety and Depression During a 3-Year Period as Well as Their Risk Factors and Prognostic Value in Postoperative Bladder Cancer Patients
Source: Front Surg. 2022 Jul 19;9:893249. doi: 10.3389/fsurg.2022.893249 (PMC9343671; doi:10.3389/fsurg.2022.893249)
Supplement: Supplementary file 3 [file Table_6_v1.docx]

**Supplementary Table 2.** Correlation between surgery type and HADS score among bladder cancer patients.

| Items | Surgery type | | | *P* value |
| --- | --- | --- | --- | --- |
|  | TURBT | Radical cystectomy by laparoscopic surgery | Radical cystectomy by open surgery |  |
| **HADS-A score, mean±SD** | |  |  |  |
| Baseline | 7.3±2.9 | 8.0±3.2 | 10.5±1.5 | 0.029 |
| 1-year | 7.9±3.1 | 7.5±3.8 | 9.0±3.3 | 0.552 |
| 2-year | 7.3±1.3 | 8.1±2.8 | 8.3±3.3 | 0.771 |
| 3-year | 8.1±2.8 | 9.3±4.0 | 13.0±NA | 0.136 |
| **HADS-D score, mean±SD** | |  |  |  |
| Baseline | 7.6±3.5 | 7.3±2.0 | 8.2±3.2 | 0.607 |
| 1-year | 7.0±1.4 | 7.2±2.8 | 7.7±3.3 | 0.675 |
| 2-year | 6.5±1.7 | 8.0±3.3 | 8.0±3.1 | 0.646 |
| 3-year | 7.8±3.1 | 8.6±3.2 | 9.0±NA | 0.578 |

HADS, Hospital Anxiety and Depression Scale; HADS-A, Hospital Anxiety and Depression Scale-Anxiety; SD, standard deviation; HADS-D, Hospital Anxiety and Depression Scale-Depression; TURBT, transurethral resection of bladder tumor; NA, not available.
